# The 2021–2022 position of Brazilian Diabetes Society on insulin therapy in type 1 diabetes: an evidence-based guideline to clinical practice

**DOI:** 10.1186/s13098-022-00949-z

**Published:** 2022-12-12

**Authors:** Wellington S. Silva Júnior, Monica Andrade Lima Gabbay, Rodrigo Nunes Lamounier, Luis Eduardo Calliari, Marcello Casaccia Bertoluci

**Affiliations:** 1 Brazilian Diabetes Society (Sociedade Brasileira de Diabetes—SBD), São Paulo, Brazil; 2 Endocrinology Discipline, Department of Medicine I, Faculty of Medicine, Center of Biological Sciences, Federal University of Maranhão (UFMA), Praça Gonçalves Dias, 21, Centro, São Luís, MA 65020-240 Brazil; 3 Federal University of São Paulo (Unifesp), São Paulo, SP Brazil; 4 Federal University of Minas Gerais (UFMG), Belo Horizonte, MG Brazil; 5 Santa Casa School of Medical Sciences, São Paulo, SP Brazil; 6 Federal University of Rio Grande do Sul (UFRGS), Porto Alegre, RS Brazil

**Keywords:** Type 1 diabetes, Insulin therapy, Insulin analogues, Management, Treatment

## Abstract

**Background:**

Insulin therapy regimens for people with type 1 diabetes (PWT1D) should mimic the physiological insulin secretion that occurs in individuals without diabetes. Intensive insulin therapy, whether by multiple daily injections (MDI) or continuous subcutaneous insulin infusion (CSII), constitutes the fundamental therapy from the initial stages of type 1 diabetes (T1D), at all ages. This review is an authorized literal translation of part of the Brazilian Diabetes Society (SBD) Guidelines 2021–2022. This evidence-based guideline supplies guidance on insulin therapy in T1D.

**Methods:**

The methods were published elsewhere in earlier SBD guidelines and was approved by the Internal Institutional Steering Committee for publication. Briefly, the Brazilian Diabetes Society indicated fourteen experts to constitute the Central Committee, designed to regulate the method review of the manuscripts, and judge the degrees of recommendations and levels of evidence. SBD Type 1 Diabetes Department drafted the manuscript selecting key clinical questions to do a narrative review using MEDLINE via PubMed, with the best evidence available, including high-quality clinical trials, metanalysis, and large observational studies related to insulin therapy in T1D, by using the Mesh terms [type 1 diabetes] and [insulin].

**Results:**

Based on extensive literature review the Central Committee defined ten recommendations. Three levels of evidence were considered: A. Data from more than one randomised clinical trial (RCT) or one metanalysis of RCTs with low heterogeneity (I^2^ < 40%). B. Data from metanalysis, including large observational studies, a single RCT, or a pre-specified subgroup analysis. C: Data from small or non-randomised studies, exploratory analysis, or consensus of expert opinion. The degree of recommendation was obtained based on a poll sent to the panellists, using the following criteria: Grade I: when more than 90% of agreement; Grade IIa if 75–89% of agreement; IIb if 50–74% of agreement, and III, when most of the panellist recommends against a defined treatment.

**Conclusions:**

In PWT1D, it is recommended to start insulin treatment immediately after clinical diagnosis, to prevent metabolic decompensation and diabetic ketoacidosis. Insulin therapy regimens should mimic insulin secretion with the aim to achieve glycemic control goals established for the age group. Intensive treatment with basal-bolus insulin therapy through MDI or CSII is recommended, and insulin analogues offers some advantages in PWT1D, when compared to human insulin. Periodic reassessment of insulin doses should be performed to avoid clinical inertia in treatment.

## Introduction

Insulin therapy regimens for people with type 1 diabetes (PWT1D) should mimic the physiological insulin secretion that occurs in individuals without diabetes. The strategy of choice is basal-bolus therapy, which should be instituted early, with multiple daily injections (MDI) therapy or with an insulin infusion pump (continuous subcutaneous insulin infusion – CSII).

At the diagnosis of PWT1D, individuals have insulin deficiency and, therefore, are highly likely to progress to diabetic ketoacidosis (DKA). Intensive insulin therapy, either by MDI or CSII, constitutes the fundamental therapy from the initial stages of the disease, at all ages [1, 2].

The insulin replacement strategy for PWT1D should mimic the physiological secretion of insulin. Traditionally, 50% of secretion is assumed as basal component, throughout the day, and the remaining 50% as prandial component, in response to meals. Basal insulins should be used for the basal component, and prandial insulins for the prandial component, preferably rapid-acting or ultra-rapid-acting analogues, with MDI or CSII (see Table 1) [2, 3].

**Table 1:** Insulin formulations available for type 1 diabetes in Brazil

Type	Generic Name	Onset*	Peak*	Duration*	References
Basal insulins					
Intermediate-acting	Neutral protamine Hagedorn (NPH)	2–4 h	4–10 h	10–18 h	[35]
Long-acting	Glargine U100	2–4 h	―	20–24 h	[35]
	Detemir	1–3 h	6–8 h	18–22 h	[36]
Ultra-long-acting	Glargine U300	6 h	―	36 h	[35]
	Degludec	< 4 h	―	42 h	[35]
Prandial insulins					
Short-acting	Regular	30–60 min	2–3 h	5–8 h	[35]
Rapid-acting	Aspart	5–15 min	30 min–2 h	3–5 h	[35]
	Lispro				[35]
	Glulisine				[35]
Ultra-rapid-acting	Ultra-rapid-acting aspart	2–5 min	1–3 h	3–5 h	[37]

^*^Variation by injection site, U/kg per injection, and within and between persons may be noted

Daily insulin requirements in T1D can be estimated from body weight, typically ranging from 0.4 U/kg/day to 1.0 U/kg/day. Larger doses may be needed during puberty, pregnancy, or infections. The prandial component is usually divided into three to four premeal boluses per day, administered 30 min before the start of a meal for regular human insulin, 20 min before the start of a meal for the rapid-acting analogues [4] and immediately before a meal for the ultra-rapid-acting analogue [5].

Table 1 summarizes the insulin formulations available for T1D in Brazil.

## Methodology

The present review is a literal authorized translation of part of the 2021–2022 Brazilian Diabetes Society (Sociedade Brasileira de Diabetes—SBD) Guidelines. The method was approved by the internal institutional Steering Committee for publication. In brief, the Brazilian Diabetes Society indicated experts to constitute the Central Committee, designed to regulate methodology, review the manuscripts, and make judgments on degrees of recommendations and levels of evidence. SBD Type 1 Diabetes Department drafted the manuscript selecting key clinical questions to make a narrative review using MEDLINE via PubMed, using the best evidence available including high-quality clinical trials, metanalysis, and large observational studies related to insulin therapy in T1D, by using the Mesh terms [type 1 diabetes] and [insulin].

### Levels of evidence

Three levels of evidence were considered: A. Data from more than 1 randomised clinical trial (RCT) or 1 metanalysis of RCTs with low heterogeneity (I^2^ < 40%). B. Data from metanalysis, including large observational studies, a single RCT, or a pre-specified subgroup analysis. C: Data from small or non-randomized studies (cross-sectional, case-control, or experimental), exploratory analyses, or consensus of expert opinion.

### Degree of recommendation

A poll was sent to all expert panelists from the Type 1 Diabetes Department and Central Committee for each defined recommendation. The frequency of responses was analyzed, and a degree of recommendation was obtained based on the following criteria: Grade I: when more than 90% of the participants agree; Grade IIa: 75–89% of the panelists agree; IIb: 50–74% of the panelists agree, and III: when the greatest part of the panelist recommends against a defined treatment. The terminology used related to the four degrees of recommendations were: I: IS RECOMMENDED; IIa: SHOULD BE CONSIDERED; IIb: MAY BE CONSIDERED; III: IT IS NOT RECOMMENDED.

## Recommendations

### R1

In PWT1D, it IS RECOMMENDED to start insulin treatment immediately after clinical diagnosis to prevent metabolic decompensation and DKA.


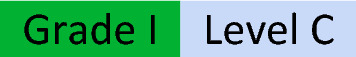


### Summary of evidence

- For ethical reasons, there are no studies comparing treating or not treating PWT1D with insulin. In situations of absolute insulin deficiency, such as T1D, DKA can be installed quickly, especially in the presence of infectious processes, and death can occur in a few hours, even after hospital admission.[1] Therefore, to prevent DKA, insulin therapy should be instituted as soon as possible after the diagnosis of T1D. If DKA is already installed, insulin therapy must follow a protocol that prioritizes the correction of hydro electrolytic and acid-base disorders.

#### R2

It IS RECOMMENDED to use insulin therapy regimens that mimic insulin secretion, with the aim to achieve glycemic control goals established for the age group, comorbidities or frailty conditions.


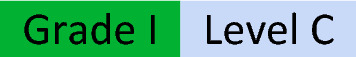


### Summary of evidence

- This panel considers, based on expert opinion, that the therapeutic regimen should be individualized according to the availability of basal and prandial insulins and with age, body weight, pubertal stage, lifestyle, individual routine, the duration and stage of diabetes, the aspect of the insulin injection site, physical activity, complications and eating habits of each patient [2]. Prescription involves knowledge about the types of insulin, sensitivity factor, insulin to carbohydrate ratio, carbohydrate counting, glycemic self-monitoring and insulin management during physical activity and stressful situations.

#### R3

In PWT1D, intensive treatment with basal-bolus insulin therapy, through MDI or CSII, IS RECOMMENDED.


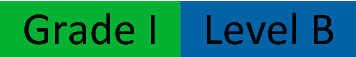


### Summary of evidence

- The Diabetes Control and Complications Trial (DCCT) [6] designed to compare intensive care vs. treatment of PWT1D in terms of the incidence of microvascular complications, has changed the treatment paradigm for T1D. Intensive therapy, with three or more daily insulin injections or with CSII, reduced and maintained glycated haemoglobin (HbA1c) vs the conventional group, reaching a nadir at six months of treatment, and about 44% of patients in the intensive group reached the target of 6% (recommended in the study). However, the incidence of severe hypoglycemia also increased significantly with intensive treatment, especially in those with lower HbA1c values [6]. The incidence of retinopathy was reduced by 76%; neuropathy, in 60%, and kidney disease from diabetes, in 39% [6]. A curvilinear relationship was found between HbA1c and the incidence of retinopathy, and a cut-off point from which further reductions in HbA1c would not bring benefit was not found [6].
- After the end of the DCCT, all patients migrated to intensive care and remained in follow-up as participants in the observational Epidemiology of Diabetes Interventions and Complications (EDIC) study [7]. Despite similar HbA1c values, patients who had been randomized to intensive treatment had a lower incidence of micro and macrovascular complications [7–10].
- These 2 studies support the recommendation that intensive insulin therapy should be the treatment of choice for T1D, as well as the choice of a target of HbA1c < 7% to reduce the incidence of chronic micro and macrovascular complications of the disease, without, however, entail a prohibitive risk of hypoglycemia.

#### R4

Long-acting insulin analogues SHOULD BE CONSIDERED for basal insulin therapy, as they induce less glycemic variability and lower incidence of nocturnal hypoglycemia compared to NPH insulin.


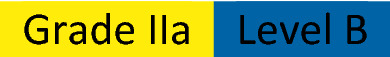


### Summary of evidence

- Long-acting analogues (glargine U100, detemir), obtained through recombinant DNA technique, entail less glycemic variability and lower risk of hypoglycemia compared to NPH insulin [11, 12]. This can be explained by the pharmacokinetic and pharmacodynamic profile of these insulins, [13, 14] which imply a more predictable action profile 11, 12. In addition, long-acting analogues are associated with a lower frequency of hypoglycemia vs NPH insulin in PWT1D [11, 15, 16].
- Children and adolescents with T1D treated with insulin glargine U100 showed better fasting glucose control for the same HbA1c, as well as a tendency to reduce severe hypoglycemia and nocturnal hypoglycemia, when compared to those treated with human NPH insulin [17].
- In a 26-week, open-label, multicenter clinical trial in PWT1D, detemir (in two daily doses) was compared to glargine U100 (in a single daily dose), both respectively associated with insulin aspart [18].These long-acting analogues have been shown to be equally effective in glycemic control, with a comparable overall risk of hypoglycemia. However, there were fewer daytime or nocturnal hypoglycemic events with insulin detemir compared to glargine U100 [18].

#### R5

Ultra-long-acting analogues MAY BE CONSIDERED for basal insulin therapy in people at increased risk for hypoglycemia, as they are associated with a lower incidence of hypoglycemia and greater flexibility.


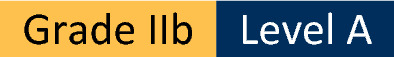


### Summary of evidence

- The efficacy and safety profile of ultra-long-acting analogues (glargine U300 and degludec) in PWT1D were evaluated in phase 3 clinical studies using insulin glargine U100 as a comparator. In EDITION 4, a six-month, open-label RCT, participants were randomised to daily insulin glargine U300 or glargine U100, both in combination with prandial insulin at each meal. Glargine U300 demonstrated non-inferiority compared to glargine U100 in reducing HbA1c from baseline. The rates of general, nocturnal and/or severe hypoglycemia were similar between the groups at the end of six months. However, there was a lower incidence of confirmed or severe nocturnal hypoglycemic events in the first eight weeks of the insulin glargine U 300 study (event ratio 0.69; 95% CI 0.53–0.91). There were no differences in results due to the time of application in the morning or at night [19].
- The BEGIN Basal-Bolus Type 1 study was a 52-week, open-label, treat-to-target clinical trial that compared the use of degludec vs. glargine U100 (both combined with prandial insulin in a multiple-dose regimen). At the end of one year, the efficacy in lowering HbA1c and the incidence of confirmed hypoglycemic events (< 56 mg/dL) were similar between the groups. Notwithstanding, the incidence of confirmed nocturnal hypoglycemia was 25% lower with degludec compared with glargine U100 (4.41 vs 5.86 episodes per patient-year of exposure; p = 0.021) [20]. It should be noted that this study excluded people with recurrent severe hypoglycemia or asymptomatic hypoglycemia [20].
- PWT1D at high risk for hypoglycemia were included in the SWITCH 1 study, [21] which compared insulin degludec vs. glargine U100 for the incidence of general, nocturnal, and/or severe hypoglycemia. Degludec promoted an 11% reduction in symptomatic hypoglycemia (p < 0.001), a 36% reduction in nocturnal hypoglycemia (p < 0.001), and a 35% reduction in severe hypoglycemia episodes (p = 0.007), compared with insulin glargine U100 [21].
- Given the pharmacokinetic and pharmacodynamic properties of the ultra-long-acting analogues, the possibility of some flexibility in the time of application of these insulins in the face of an unexpected event is admitted without prejudice to glycemic control and ensuring greater convenience. Although this has not been effectively tested for insulin glargine U300 in PWT1D, insulin degludec has been tested in the BEGIN Flex T1 study [22]. In this 26-weeks open-label, treat-to-target clinical trial, degludec administered in a forced flexible regimen (minimum interval of 8 h and maximum interval of 40 h between doses) was shown to be safe and non-inferior to the use of insulin degludec or glargine U100 given at fixed times [22].

#### R6

For most individuals with T1D, the use of rapid-acting or ultra-rapid-acting insulin analogues in the basal-bolus regimen IS RECOMMENDED to reduce the risk of hypoglycemia.


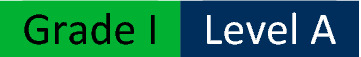


### Summary of evidence

- Bolus insulin or prandial insulin is an indispensable component of basal-bolus therapy. Short-acting (regular) subcutaneous human insulin, rapid-acting insulin analogues (lispro, aspart, and glulisine), and ultra-rapid-acting insulin analogue (ultra-rapid-acting aspart) are currently available.
- A recent meta-analysis carried out by the SBD included eighteen RCTs comparing rapid-acting insulin analogues vs. regular insulin in PWT1D. There was a 32% reduction in episodes of severe hypoglycemia [RR 0.68 (95% CI 0.60–0.77)] and a 45% reduction in nocturnal hypoglycemia episodes [RR 0.55 (95% CI 0.40–0.76)] in favor of the rapid-acting insulin analogues. It is noteworthy the great heterogeneity of the studies included in the meta-analysis and the fact that, in many of them, episodes of hypoglycemia were counted as adverse events and not as primary outcomes [23].
- In another systematic review and meta-analysis of RCTs with the same objective, the median incidences of severe hypoglycemia in the T1D population were 21.8 episodes and 46.1 episodes per 100 person-years for the rapid-acting analogues and for the regular human insulin, respectively [24].
- In young children, lispro insulin before dinner vs. regular human insulin reduced the risk of nocturnal hypoglycemia without compromising HbA1c in an open-label crossover study [25].

#### R7

It IS RECOMMENDED that prandial insulin be administered before each meal, as it is superior to post-meal injection for the best postprandial glycemic control.


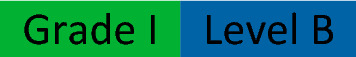


### Summary of evidence

- The optimal time to administer the rapid‐acting insulin analogues is 15–20 min before meals, once it reduces postprandial glycemic excursions compared with the application immediately before the meal or up to 20 min later [4, 26]. In young children, when there is doubt about the total intake of programmed carbohydrates, the administration of rapid-acting insulin analogues can be performed after meals, proving to be as effective as regular human insulin administered before meals [27].
- The direct comparison between the rapid-acting insulin analogues did not show differences in the effective control of postprandial glycemic excursions promoted by these agents. In addition, in the presence of hyperglycemia, it is recommended that rapid-acting insulin analogues are always administered in advance of the meal.

#### R8

When there is uncertainty regarding food intake and the need for flexible hours, ultra-rapid-acting insulin MAY BE CONSIDERED for administration after meal, as it offers advantages over rapid-acting analogues.


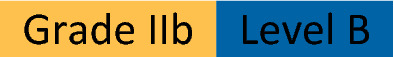


### Summary of evidence

- The ultra-rapid aspart analogue showed superiority in the control of postprandial glycemia when administered before the meal and non-inferiority when administered within 20 min after the start of meals vs. the rapid-acting analogue administered before the meal [28]. The pharmacokinetic behaviour of ultra-rapid insulin aspart in PWT1D using CSII was even more pronounced. There was a peak of action 25 min earlier than that of the rapid-acting insulin aspart and there was a reduction in postprandial glycemia at 30 min, 1 h and 2 h after the meal test [29].
- The ultra-rapid aspart can be used immediately before meals. It is approved for people from one year of age.

#### R9

CSII IS RECOMMENDED as an effective therapeutic option to achieve adequate glycemic control when this is not possible to be achieved with MDI.


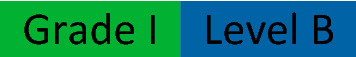


### Summary of evidence

- CSII is considered the gold standard in intensive care of T1D, but it requires monitoring by a trained team [30].
- When comparing CSII with MDI, the kind of glucose monitoring associated with the treatment seems to influence the results. In a real-world study, ninety-four adults with T1D were followed up for three years, divided into four groups: two with CSII and two with MDI, and for each modality there was a group with continuous glucose monitoring (CGM) and another with capillary blood glucose. The MDI + CGM regimen had similar results to the CSII + CGM, with a better cost–benefit ratio, and the use of CGM was superior to capillary blood glucose in reducing hypoglycemia and HbA1c. It is worth mentioning that the CSII used in the study was not the hybrid closed loop one [31].
- In a special population (young children), CSII may be the only option, as the daily insulin requirement is very low and doses below 0.5 U are needed, which is unfeasible in MDI therapy. For this reason, the SBD recommends treatment with CSII as the main form of therapy for young children.

#### R10

Periodic reassessment of insulin doses IS RECOMMENDED to avoid clinical inertia in treatment.


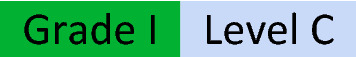


### Summary of evidence

- The total daily dose of insulin (DDI) will depend on time of diagnosis, age, weight and stage of puberty. In general, young children need lower doses of insulin per kg compared to adults and older children, while adolescents use higher doses than other age groups. Infants usually need 0.3 U/kg/day to 0.5 U/kg/day; prepubertal and adults, from 0.7 U/kg/day to 1.0 U/kg/day; pubescents, from 1.0 U/kg/day to 2.0 U/kg/day. It is worth mentioning that, during the remission phase, the required DDI is less than 0.5 U/kg/day. On the other hand, on sick days, the need for insulin increases.
- Basal insulin requirement is typically 30% to 50% of the DDI, and the rest is reserved for bolus insulin, split before meals. In infants, care should be taken to maintain the basal insulin dose around 30% of the DDI, due to the great irregularity in the feeding rhythm and energy expenditure. In adolescents, the baseline requirement may be 50% to 55%. The distribution of bolus doses varies greatly from person to person. These doses should be adjusted according to pre-meal blood glucose, the foods that will be ingested, physical activity and situations related to health events [32].
- Basal insulin dose adjustment is performed according to fasting blood glucose, and bolus insulin doses are adjusted according to pre-prandial and two to three hours post-meal blood glucose, considering the sensitivity factor (SF) and the current insulin to carbohydrate ratio. The SF represents how much 1 U of prandial insulin lowers blood glucose, and it is suggested by the result of dividing 2000 by the DDI. The division of 2100 by the DDI can be used in infants and 1800 by the DDI, in adults.
- The insulin to carbohydrate ratio is obtained by dividing 400 by the DDI, and corresponds to the amount of carbohydrate that can be consumed in such a way that blood glucose is not altered after the administration of 1 U of insulin [33, 34].

Table 2 summarizes the final recommendations on insulin therapy in T1D in Brazil.

**Table 2:**
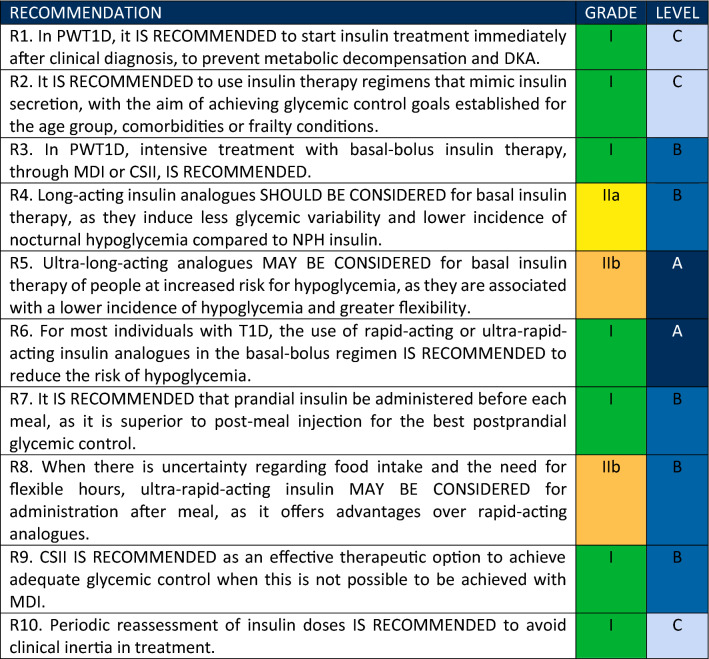
Final recommendations

## Data Availability

Not applicable.
